# Smoke-Free Rules and Secondhand Smoke Exposure in Vehicles among U.S. Adults—National Adult Tobacco Survey, 2009–2010 and 2013–2014

**DOI:** 10.3390/ijerph13111048

**Published:** 2016-10-26

**Authors:** Judy Kruger, Amal Jama, Michelle Kegler, Carissa Baker Holmes, Sean Hu, Brian King

**Affiliations:** 1 Office on Smoking and Health, National Center for Chronic Disease Prevention and Health Promotion, Centers for Disease Control and Prevention, Atlanta, GA 30329, USA; ipz3@cdc.gov (C.B.H.); fik4@cdc.gov (S.H.); iyn3@cdc.gov (B.K.); 2 DB Consulting Group, Atlanta, GA 30329, USA; amal.o.jama@gmail.com; 3 Rollins School of Public Health, Emory University, Atlanta, GA 30322, USA; mkegler@emory.edu

**Keywords:** tobacco products, secondhand smoke exposure, vehicles, policy

## Abstract

In the United States (U.S.), secondhand smoke (SHS) exposure causes more than 41,000 deaths among nonsmoking adults annually. Adoption of smoke-free laws in public areas has increased, but private settings such as vehicles remain a source of SHS exposure. This study assessed change in voluntary smoke-free vehicle rules and SHS exposure in personal vehicles among U.S. adults between two periods, 2009–2010 and 2013–2014, using data from the National Adult Tobacco Survey (NATS). NATS is a national landline and cellular telephone survey of non-institutionalized adults aged ≥18 years in the 50 U.S. states and the District of Columbia. We assessed percentage change in the prevalence of smoke-free vehicle rules among all adults and SHS exposure in vehicles among nonsmoking adults, overall, by sociodemographic factors (sex, age, race/ethnicity, education, marital status, annual household income, U.S. region), and by cigarette smoking status. During 2009–2010 to 2013–2014, the percentage of adults with a 100% smoke-free vehicle rule increased from 73.6% to 79.5% (% change = +8.0%; *p* < 0.05). Among nonsmokers, SHS exposure in vehicles in the previous 7 days decreased from 9.2% to 8.2% (% change = −10.9%; *p* < 0.05). Smoke-free rules in private settings such as vehicles, in coordination with comprehensive smoke-free policies in indoor public settings, can help reduce SHS exposure and promote smoke-free norms.

## 1. Introduction

Secondhand smoke (SHS) is a mixture of the smoke produced by the burning end of a tobacco product and the smoke exhaled by smokers [1]. Exposure to SHS causes heart disease and lung cancer in nonsmoking adults and sudden infant death syndrome, acute respiratory infections, ear problems, and more severe asthma in children [1,2]. Each year, SHS exposure causes an estimated 3000 lung cancer deaths and more than 46,000 heart disease deaths among U.S. adult nonsmokers [2]. Moreover, the U.S. Surgeon General has concluded that there is no risk-free level of SHS and that eliminating smoking in indoor spaces is the only effective way to fully protect nonsmokers from the adverse effects of SHS exposure [1,2]. SHS exposure has declined over the past two decades [1], particularly due to comprehensive smoke-free laws in indoor public areas, including worksites, restaurants, and bars; however, many U.S. nonsmokers are exposed to SHS in private settings, including vehicles [3]. Studies have found that passengers riding in personal vehicles with consistently enforced 100% smoke-free vehicle rules have lower rates of SHS exposure in vehicles than those without such rules [4,5].

Vehicles represent an important environment for exposure to SHS. Smoking in a vehicle can lead to elevated levels of fine particle air pollution and airborne nicotine within the vehicle [6,7,8,9]. Moreover, youth exposed to SHS in vehicles may be at an increased risk for adverse respiratory health effects when compared to unexposed youth, including current and persistent wheeze, hay fever symptoms, and decreased lung function [10,11]. Smoking in vehicles also appears to occur at higher rates among socioeconomically disadvantaged populations, and thus, may contribute to inequalities in SHS-attributable health outcomes [10,12].

To date, increases have occurred in public smoke-free laws and voluntary smoke-free home rules [13]. As of October 2016, seven U.S. states and one territory have implemented legislation prohibiting smoking in personal vehicles where children less than a specified age are present [14]. However, changes in voluntary smoke-free rules and SHS exposure in vehicles is uncertain. To address this gap, this study used the most recently available data from the National Adult Tobacco Survey (2009–2010 and 2013–2014) to assess self-reported changes in smoke-free vehicle rules and SHS exposure in vehicles among U.S. adults.

## 2. Materials and Methods

### 2.1. Sample

Data came from the 2009–2010 and 2013–2014 National Adult Tobacco Surveys (NATS). NATS is a stratified, random-digit dialed, telephone survey of non-institutionalized, civilian U.S. adults aged ≥18 years or older; a complete description of NATS methodology is available elsewhere [15]. The sample was designed to yield representative national and state data from households in the 50 U.S. states and the District of Columbia. Each state was divided into strata by phone type. For landline numbers, one adult was randomly selected from each eligible household. Alternatively, adults who only used cellular phones were selected through screening of a sample of cellular phone numbers. This study used secondary data, and thus, was exempt from human subjects review. In total, 118,581 interviews were completed during 2009–2010 and 75,233 were completed during 2013–2014. The overall response rate was 37.6% in 2009–2010 (landline = 40.4%, cellular 24.9%) and 36.1% in 2013–2014 (landline = 47.6%, cellular = 17.1%).

### 2.2. Measurements

The presence of smoke-free vehicle rules and SHS exposure in vehicles were assessed using the same questions on both the 2009–2010 and 2013–2014 NATS. Smoke-free vehicle rules were assessed by the question, “Not counting motorcycles, in the vehicles that you or family members who live with you own or lease, is smoking “always allowed (in all vehicles)”, “sometimes allowed in at least one vehicle”, or “never allowed in any vehicle”?” Respondents who selected “never allowed in any vehicle” were classified as having a 100% smoke-free vehicle rule. Exposure to SHS in a vehicle was assessed by the question, “During the past 7 days, (that is, since last (TODAY’S DAY OF WEEK)), on how many days did you ride in a vehicle where someone other than you was smoking tobacco?” Response options ranged from “0” through “7”. Respondents who answered “1” through “7” were classified as being exposed to SHS in a vehicle within the previous 7 days, while those who answered “0” were classified as not being exposed.

Assessed sociodemographic characteristics included: sex, age, race/ethnicity, education, marital status, annual household income, and U.S. Census region. Cigarette smoking status was classified as current smokers (smoked ≥100 cigarettes in lifetime and now smoked “every day” or “somedays”), former smokers (smoked ≥100 cigarettes in lifetime and now smoked “not at all”) and never smokers (did not smoke ≥100 cigarettes in lifetime). For the purposes of analyzing SHS exposure, nonsmokers consisted of former and never cigarette smokers.

### 2.3. Statistical Analysis

Data were analyzed using SAS-Callable SUDAAN 10 (RTI International, Research Triangle Park, NC, USA) and weighted to adjust for the differential probabilities of selection and response. For 2009–2010 and 2013–2014, national prevalence estimates and Wald 95% confidence intervals were calculated overall and by sociodemographics and cigarette smoking status. Estimates of SHS exposure were stratified by smoke-free vehicle rule status because a statistically significant association was observed between SHS exposure and smoke-free vehicle rule status. Differences between groups were assessed using two-sided *t*-tests (*p* < 0.05). Relative percent change in smoke-free rules and SHS exposure during 2009–2010 to 2013–2014 was also assessed. For all analyses, smoke-free vehicle rules were assessed among all adults, while SHS exposure was assessed among nonsmokers only.

## 3. Results

### 3.1. Overall Sample

Sample characteristics in the 2009–2010 NATS were as follows: male (48.6%), female (51.4%); 18–24 years of age (13.1%), 25–44 years (36.6%), 45–64 years (33.6%), ≥65 years (16.7%); white non-Hispanic (68.8%), black non-Hispanic (11.7%), other non-Hispanic (6.4%), and Hispanic (13.2%) (data not shown). Sample characteristics in the 2013–2014 NATS were as follows: male (48.2%), female (51.8%); 18–24 years of age (11.9%), 25–44 years (34.9%), 45–64 years (34.6%), ≥65 years (18.6%); white non-Hispanic (65.4%), black non-Hispanic (11.8%), other non-Hispanic (7.9%), and Hispanic (15.0%) (data not shown).

### 3.2. Smoke-Free Vehicle Rules

During 2009–2010 to 2013–2014, the percentage of all adults with a 100% smoke-free vehicle rule increased from 73.6% to 79.5% (relative % change = +8.0%; *p* < 0.05) (Table 1). Increases occurred in the percentage of adults with 100% smoke-free vehicle rules across all assessed sociodemographic groups (*p* < 0.05).

### 3.3. Secondhand Smoke Exposure

During 2009–2010 to 2013–2014, the overall percentage of nonsmoking adults exposed to SHS in a vehicle within the previous 7 days decreased from 9.2% to 8.2% (relative % change = −10.9%; *p* < 0.05) (Table 2). Significant declines in exposure were observed for black non-Hispanics, Hispanics, those married or living with a partner, those with an unspecified annual household income, and those residing in the Midwest or South (*p* < 0.05).

Among those with 100% smoke-free vehicle rules, the overall percentage of nonsmoking adults exposed to SHS in a vehicle within the previous 7 days did not change significantly (Table 2). Among those with 100% smoke-free rules, significant increases in exposure were observed for white non-Hispanics, high school graduates, those single/separated/divorced or widowed, and those with incomes of $20,000–$49,999 (*p* < 0.05).

Among those with no smoke-free vehicle rules, the overall percentage of nonsmoking adults exposed to SHS in a vehicle within the previous 7 days did not change significantly (Table 2).

## 4. Discussion

This study found that progress has been made in adopting voluntary smoke-free vehicle rules in the U.S. over a relatively short period of time; 79.5% of U.S. adults (about 8 in 10 adults) reported having a 100% smoke-free vehicle rule in 2013–2014, representing an 8% relative percent increase from 2009–2010. However, although the adoption of smoke-free policies has increased, vehicles remain a source of secondhand smoke exposure for some nonsmoking adults. Moreover, respondents with a smoke-free vehicle rule experienced decreased SHS exposure during the assessed period, while those without a smoke-free rule saw no change. Passengers riding in personal vehicles with consistently enforced 100% smoke-free vehicle rules are more likely to have lower rates of exposure to airborne nicotine and SHS compared to those without smoke-free rules [4]. However, consistent with previous research [16], this study found that overall, many individuals remain exposed to SHS in vehicles. More specifically, nearly 1 in 10 nonsmoking U.S. adults (8.2%) were still exposed to SHS in a vehicle during 2013–2014.

SHS exposure declined among: both sexes; all age groups; non-Hispanic whites, non-Hispanic blacks and Hispanics; those with 0–12 years of education, a general educational diploma, high school diploma or some college; all marital status groups; all household income groups; and U.S. Census regions. Of note, SHS exposure in a vehicle among nonsmoking adults declined overall during the assessed period, but trended upward when stratified by smoke-free rule status. This finding may be the result of Simpson’s Paradox [17]; that is, due to the fact that the proportion of people who had a smoke-free vehicle rule in 2013–2014 was larger than in 2009–2010.

Voluntary smoke-free vehicle rules have been shown to help reduce the number of people exposed to SHS [18,19]. In addition, policies prohibiting smoking in vehicles in which children are present, in coordination with comprehensive smoke-free policies in indoor public settings, may help reduce SHS exposure and promote smoke-free norms. To date, some states and one U.S. territory have implemented legislation prohibiting smoking in personal vehicles where children less than a specified age are present, including Arkansas, California, Louisiana, Maine, Oregon, Puerto Rico, Utah, and Vermont [14]. Research suggests that these laws may promote the adoption of voluntary smoke-free vehicles rules; for example, the prevalence of voluntary smoke-free vehicles rules among Maine adults was significantly higher after the passage of a statewide smoke-free vehicle law [20]. These findings underscore the importance of enhanced and sustained efforts to increase awareness of the dangers of SHS exposure and to encourage the adoption of voluntary smoke-free rules in vehicles [21].

Strengths of this study include the use of data from a large, nationally representative sample to investigate changes over time. However, the findings are subject to some limitations. First, the use of self-reported tobacco use and assessment of smoke-free rules could have introduced bias. Second, data were cross-sectional, and thus, it was not possible to assess the temporal relationship between smoke-free vehicle rules and SHS exposure. Third, because of a difference in the distribution of landline and cellular phone response rates, we calculated national and state estimates differently to prevent large variances of survey estimates. Fourth, the questionnaire did not include variables that might affect smoke-free rules in vehicles, such as the presence of children in the vehicle.

## 5. Conclusions

To our knowledge, this is the first national study to assess changes in smoke-free vehicle rules and SHS exposure in vehicles over time. The findings indicate that smoke-free vehicle rules have increased among U.S. adults, but some remain exposed to SHS in this environment. Voluntary smoke-free vehicle rules, in coordination with comprehensive smoke-free policies in indoor public areas, can help reduce SHS exposure [1].

## Figures and Tables

**Table 1:** Percentage of U.S. adults who reported having a 100% smoke-free vehicle rule, by selected sociodemographic characteristics and cigarette smoking status—National Adult Tobacco Survey, 2009–2010 and 2013–2014.

Characteristics	100% Smoke-Free Vehicle Rule ^a^		
	Overall 2009–2010 (*n* = 116,914)	Overall 2013–2014 (*n* = 68,595)	Relative % Change (2009–2010 to 2013–2014)
	% (95% CI)	% (95% CI)	%
**Overall**	73.6 (73.1–74.2)	79.5 (79.0–79.9)	+8.0 **^b^**
**Sex**			
Male	71.0 (70.1–71.9)	77.2 (76.5–77.8)	+8.7 **^b^**
Female	76.2 (75.5–76.8)	81.7 (81.1–82.3)	+7.2 **^b^**
**Age (years)**			
18–24	61.1 (59.0–63.2)	71.0 (69.3–72.6)	+16.2 **^b^**
25–44	72.3 (71.3–73.3)	78.0 (77.1–78.8)	+7.8 **^b^**
45–64	74.6 (73.8–75.4)	79.3 (78.6–80.0)	+6.3 **^b^**
≥65	84.7 (83.8–85.6)	87.6 (87.0–88.2)	+3.5 **^b^**
**Race/ethnicity**			
White, non-Hispanic	72.6 (72.0–73.1)	78.4 (77.9–79.0)	+8.1 **^b^**
Black, non-Hispanic	71.7 (69.8–73.6)	78.0 (76.6–79.4)	+8.8 **^b^**
Other, non-Hispanic **^c^**	76.1 (73.9–78.3)	80.4 (78.7–82.1)	+5.6 **^b^**
Hispanic	79.1 (76.8–81.3)	85.4 (84.1–86.7)	+8.0 **^b^**
**Education**			
0–12 years (no diploma)	65.8 (63.6–68.0)	71.4 (69.6–73.2)	+8.5 **^b^**
General educational diploma	49.0 (44.9–53.2)	57.9 (54.3–61.6)	+18.1 **^b^**
High school graduate	69.1 (68.0–70.3)	74.7 (73.7–75.7)	+8.0 **^b^**
Some college (no degree)	71.5 (70.2–72.7)	76.7 (75.6–77.9)	+7.4 **^b^**
Associate degree	74.1 (72.9–75.4)	79.5 (78.4–80.6)	+7.2 **^b^**
Undergraduate degree	84.8 (84.0–85.6)	89.1 (88.5–89.7)	+5.0 **^b^**
Graduate degree	88.6 (87.7–89.5)	92.1 (91.4–92.8)	+4.0 **^b^**
**Marital status**			
Married/living with partner	77.2 (76.5–77.9)	82.6 (82.1–83.2)	+7.0 **^b^**
Single/separated/divorced/widowed	68.5 (67.5–69.5)	74.9 (74.2–75.7)	+9.4 **^b^**
**Annual household income**			
<$20,000	63.8 (61.9–65.7)	69.0 (67.3–70.7)	+8.1 **^b^**
$20,000–$49,999	68.0 (66.9–69.1)	74.0 (73.0–74.9)	+8.8 **^b^**
$50,000–$99,999	76.3 (75.3–77.2)	81.2 (80.4–82.0)	+6.5 **^b^**
≥$100,000	83.8 (82.7–84.9)	88.3 (87.5–89.1)	+5.4 **^b^**
Unspecified	78.8 (77.1–80.5)	81.6 (80.6–82.6)	+3.6 **^b^**
**U.S. Census region ^d^**			
Northeast	75.0 (73.8–76.3)	81.5 (80.5–82.6)	+8.7 **^b^**
Midwest	68.3 (67.2–69.4)	74.9 (73.8–75.9)	+9.6 **^b^**
South	71.8 (70.9–72.7)	77.4 (76.7–78.2)	+7.8 **^b^**
West	80.6 (79.4–81.8)	85.6 (84.8–86.3)	+6.2 **^b^**
**Cigarette smoking status ^e^**			
Current smoker	27.0 (25.6–28.4)	31.2 (29.9–32.6)	+15.7 **^b^**
Former smoker	81.7 (80.8–82.6)	85.1 (84.4–85.8)	+4.2 **^b^**
Never smoker	86.3 (85.7–86.9)	90.7 (90.3–91.2)	+5.2 **^b^**

Abbreviation: CI = confidence interval; **^a^** Defined as a response of “never allowed in any vehicle” to the question: “Not counting motorcycles, in the vehicles that you or family members who live with you own or lease, is smoking “always allowed in all vehicles”, “sometimes allowed in at least one vehicle”, or “never allowed in any vehicles”?” **^b^** Statistically significant difference in relative percent change during 2009–2010 to 2013–2014 (*p* < 0.05). A “+” indicates an increase in the magnitude of the estimate during 2009–2010 to 2013–2014. **^c^** Other non-Hispanics, refers to Asian, American Indian/Alaska Native Non-Hispanic, multiracial and other. **^d^** Northeast: Connecticut, Maine, Massachusetts, New Jersey, New Hampshire, New York, Pennsylvania, Rhode Island, and Vermont; Midwest: Illinois, Indiana, Iowa, Kansas, Michigan, Minnesota, Missouri, Nebraska, North Dakota, Ohio, South Dakota, and Wisconsin; South: Alabama, Arkansas, Delaware, District of Columbia, Florida, Georgia, Kentucky, Louisiana, Maryland, Mississippi, North Carolina, Oklahoma, South Carolina, Tennessee, Texas, Virginia, and West Virginia; *West*: Alaska, Arizona, California, Colorado, Hawaii, Idaho, Montana, Nevada, New Mexico, Oregon, Utah, Washington, and Wyoming. **^e^** Current smoker refers to a respondent who reported smoking at least 100 cigarettes during their lifetime, and now smoked “every day” or “some days”; former smoker refers to a respondent who smoked ≥100 cigarettes in their lifetime and now smoked “not at all”; and never smokers refers to a respondent who reported that they did not smoke ≥100 cigarettes in lifetime.

**Table 2:** Percentage of nonsmoking U.S. adults who reported exposure to secondhand smoke in a vehicle in which they rode during the previous 7 days, overall and by smoke-free vehicle rule status and selected sociodemographic characteristics—National Adult Tobacco Survey, 2009–2010 and 2013–2014.

Characteristics	Secondhand Smoke Exposure in a Vehicle among Nonsmoking Adults ^a^								
	Overall ^b^			100% Smoke-Free Vehicle Rule ^c^			No 100% Smoke-Free Vehicle Rule ^d^		
	2009–2010 (*n* = 101,426) % (95% CI)	2013–2014 (*n* = 60,076) % (95% CI)	Relative % Change	2009–2010 (*n* = 88,291) % (95% CI)	2013–2014 (*n* = 53,631) % (95% CI)	Relative % Change	2009–2010 (*n* = 11,845) % (95% CI)	2013–2014 (*n* = 5539) % (95% CI)	Relative % Change
**Total**	9.2 (8.8–9.6)	8.2 (7.9–8.5)	−10.9 **^g^**	4.4 (4.0–4.8)	4.7 (4.4–5.0)	+6.8	36.2 (34.4–38.0)	37.3 (35.5–39.1)	+3.0
**Sex**									
Male	10.7 (10.0–11.4)	9.7 (9.2–10.3)	−9.3	5.3 (4.7–5.9)	5.7 (5.2–6.1)	+7.5	37.6 (34.9–40.4)	38.6 (36.1–41.1)	+2.7
Female	7.9 (7.4–8.4)	6.9 (6.4–7.3)	−12.7	3.6 (3.2–4.0)	3.8 (3.4–4.2)	+5.6	34.7 (32.4–37.0)	35.8 (33.3–38.3)	+3.2
**Age (years)**									
18–24	21.6 (19.5–23.6)	19.5 (17.9–21.2)	−9.7	11.0 (9.3–12.8)	11.7 (10.2–13.3)	+6.4	48.6 (43.8–53.4)	51.0 (46.5–55.4)	+4.9
25–44	9.5 (8.8–10.3)	8.7 (8.1–9.4)	−8.4	4.9 (4.2–5.5)	5.4 (4.8–5.9)	+10.2	37.1 (33.9–40.3)	37.8 (34.4–41.2)	+1.9
45–64	7.2 (6.6–7.8)	6.7 (6.2–7.2)	−6.9	3.2 (2.7–3.7)	3.4 (3.1–3.8)	+6.3	32.1 (29.6–34.7)	34.7 (31.8–37.6)	+8.1
≥65	4.0 (3.6–4.5)	3.9 (3.5–4.2)	−2.5	1.9 (1.5–2.2)	2.1 (1.8–2.4)	+10.5	22.0 (18.9–25.0)	23.3 (20.6–26.1)	+6.0
**Race/ethnicity**									
White-non-Hispanic	8.2 (7.8–8.7)	7.7 (7.3–8.1)	−6.1	3.4 (3.1–3.7)	4.0 (3.7–4.3)	+17.6 **^g^**	35.2 (33.3–37.0)	36.4 (34.4–38.5)	+3.4
Black-non-Hispanic	13.6 (11.9–15.3)	11.1 (9.8–12.4)	−18.4 **^g^**	6.7 (5.4–8.0)	7.1 (6.0–8.3)	+6.0	44.7 (38.8–50.6)	41.4 (35.9–46.9)	−7.4
Other-non-Hispanics **^e^**	8.3 (6.6–9.9)	8.4 (7.1–9.6)	0	4.5 (3.1–5.8)	5.4 (4.3–6.5)	+20.0	33.8 (26.4–41.2)	36.4 (29.4–43.4)	+7.7
Hispanic	11.1 (9.3–13.0)	8.7 (7.5–9.9)	−21.6 **^g^**	7.4 (5.7–9.0)	5.8 (4.7–6.9)	−21.6	34.8 (27.3–42.3)	40.5 (33.9–47.1)	−16.4
**Education**									
0–12 years (no diploma)	15.0 (13.0–17.0)	12.4 (10.8–14.1)	−17.3	8.4 (6.6–10.3)	7.4 (6.0–8.9)	−11.9	44.7 (38.4–51.1)	43.7 (37.5–49.9)	−1.6
General educational diploma	19.1 (14.5–23.6)	16.0 (12.4–19.6)	−16.2	9.2 (4.9–13.5)	7.2 (4.3–10.1)	−21.7	51.1 (39.9–62.3)	52.0 (41.1–62.8)	+1.8
High school graduate	11.8 (10.9–12.7)	11.2 (10.4–12.0)	−5.1	5.3 (4.6–6.0)	6.3 (5.6–7.0)	+18.9 **^g^**	40.2 (36.9–43.6)	40.9 (37.5–44.2)	+1.7
Some college (no degree)	10.0 (9.0–11.0)	9.1 (8.2–10.0)	−9.0	4.2 (3.5–5.0)	5.0 (4.2–5.7)	+19.0	37.4 (33.5–41.2)	38.6 (34.6–42.7)	+3.2
Associate degree	8.3 (7.4–9.2)	8.3 (7.5–9.1)	0	4.1 (3.4–4.8)	4.9 (4.3–5.6)	+19.5	33.0 (28.9–37.0)	35.7 (31.5–39.9)	+8.2
Undergraduate degree	4.4 (3.9–4.9)	4.4 (4.0–4.9)	0	2.3 (1.9–2.7)	2.8 (2.4–3.2)	+21.7	23.0 (19.9–26.2)	26.2 (22.7–29.7)	+13.9
Graduate degree	2.9 (2.3–3.4)	2.8 (2.3–3.2)	−3.4	1.6 (1.1–2.0)	1.7 (1.4–2.1)	+6.3	17.7 (14.0–21.4)	21.1 (16.3–25.8)	+19.2
**Marital status**									
Married/living with partner	7.1 (6.7–7.6)	6.1 (5.8–6.5)	−14.1 **^g^**	3.4 (3.0–3.8)	3.3 (2.9–3.6)	−2.9	32.8 (30.6–35.1)	34.8 (32.4–37.2)	+6.1
Single/separated/divorced/widowed	12.4 (1.5–13.2)	11.5 (10.8–12.1)	−7.3	5.9 (5.3–6.6)	7.1 (6.5–7.7)	+20.3 **^g^**	39.8 (37.0–42.6)	39.5 (36.9–42.2)	−0.8
**Annual household income**									
<$20,000	13.9 (12.0–15.7)	12.5 (11.0–14.0)	−10.1	7.4 (5.6–9.1)	7.5 (6.2–8.8)	+1.4	42.5 (36.9–48.0)	39.8 (34.3–45.2)	−6.4
$20,000–$49,999	11.7 (10.8–12.5)	11.6 (10.8–12.5)	−0.9	5.1 (4.5–5.8)	6.7 (6.0–7.4)	+31.4 **^g^**	40.6 (37.5–43.7)	42.8 (39.5–46.1)	+5.4
$50,000–$99,999	7.6 (7.0–8.3)	7.3 (6.6–7.9)	−3.9	3.8 (3.3–4.3)	4.3 (3.7–4.8)	+13.2	30.7 (27.8–33.6)	33.8 (30.6–37.0)	+10.1
≥$100,000	5.0 (4.3–5.8)	4.5 (3.9–5.0)	−10.0	2.5 (1.9–3.0)	2.6 (2.1–3.0)	+4.0	26.5 (21.7–31.2)	30.2 (25.5–34.9)	+14.0
Unspecified	9.1 (7.7–10.5)	7.2 (6.4–7.9)	−20.9 **^g^**	4.6 (3.4–5.7)	3.9 (3.3–4.5)	−15.2	40.4 (33.8–46.9)	36.2 (31.8–40.5)	−10.4
**U.S. Census region ^f^**									
Northeast	8.2 (7.3–9.1)	7.1 (6.4–7.9)	−13.4	4.2 (3.5–4.9)	4.5 (3.9–5.2)	+7.1	30.9 (26.9–34.9)	29.8 (25.6–34.0)	−3.6
Midwest	11.0 (10.0–11.9)	9.6 (8.8–10.4)	−12.6 **^g^**	4.4 (3.7–5.1)	5.0 (4.4–5.6)	+13.6	39.2 (36.1–42.4)	38.4 (34.9–41.9)	−2.0
South	10.7 (9.9–11.4)	9.4 (8.8–10.0)	−12.7 **^g^**	5.1 (4.5–5.7)	5.1 (4.5–5.6)	0	40.0 (37.0–43.0)	42.7 (39.9–45.5)	+6.8
West	6.2 (5.3–7.0)	6.0 (5.4–6.6)	−3.2	3.4 (2.6–4.1)	3.9 (3.3–4.5)	+14.7	29.2 (24.6–33.8)	31.4 (27.2–35.6)	+7.5

Abbreviation: CI = confidence interval. **^a^** Defined as a response between “1” and “7” to the question: “During the past 7 days, on how many days did you ride in a vehicle where someone other than you was smoking tobacco”? Non-smoking adults include both former and never smokers. Former smoker refers to a respondent who smoked ≥100 cigarettes in their lifetime and now smoked “not at all”; and never smoker refers to a respondent who reported that they did not smoke ≥100 cigarettes in lifetime. **^b^** Secondhand smoke exposure in a vehicle among nonsmoking adults declined overall, but trended upward when stratified by smoke-free rule status. This pattern may be the result of Simpson’s Paradox. This finding may be due to the fact that the proportion of people who had a smoke-free vehicle rule in 2013–2014 is larger than in 2009–2010. **^c^** Defined as a response of “never allowed in any vehicle” to the question: “Not counting motorcycles, in the vehicles that you or family members who live with you own or lease, is smoking “always allowed in all vehicles”, “sometimes allowed in at least one vehicle”, or “never allowed in any vehicles”?” **^d^** Defined as a response of “always allowed in all vehicles” or “sometimes allowed in at least one vehicle” to the question: “Not counting motorcycles, in the vehicles that you or family members who live with you own or lease, is smoking “always allowed in all vehicles”, “sometimes allowed in at least one vehicle”, or “never allowed in any vehicles”?” For 2009–2010 and 2013–2014 there were 1290 and 906 missing values for the smoke-free vehicle rule question respectively. Missing values were excluded from the analysis. **^e^** Other non-Hispanics, refers to Asian, American Indian/Alaska Native Non-Hispanic, multiracial and other. **^f^** Northeast: Connecticut, Maine, Massachusetts, New Jersey, New Hampshire, New York, Pennsylvania, Rhode Island, and Vermont; Midwest: Illinois, Indiana, Iowa, Kansas, Michigan, Minnesota, Missouri, Nebraska, North Dakota, Ohio, South Dakota, and Wisconsin; South: Alabama, Arkansas, Delaware, District of Columbia, Florida, Georgia, Kentucky, Louisiana, Maryland, Mississippi, North Carolina, Oklahoma, South Carolina, Tennessee, Texas, Virginia, and West Virginia; West: Alaska, Arizona, California, Colorado, Hawaii, Idaho, Montana, Nevada, New Mexico, Oregon, Utah, Washington, and Wyoming. **^g^** Statistically significant difference in relative percent change during 2009–2010 to 2013–2014 (*p* < 0.05). A “+” indicates an increase in the magnitude of the estimate during 2009–2010 to 2013–2014, while a “–” indicates a decrease in the magnitude of the estimate during this period.

## Abbreviations

The following abbreviations are used in this manuscript:

NATS	National Adult Tobacco Survey
SHS	Secondhand smoke
U.S.	United States
